# Intramuscular Expression of Plasmid-Encoded FVII-Fc Immunoconjugate for Tumor Immunotherapy by Targeting Tumoral Blood Vessels and Cells

**DOI:** 10.3389/fonc.2021.638591

**Published:** 2021-05-24

**Authors:** Liping Ma, Guanru Wang, Sijia Liu, Feng Bi, Ming Liu, Gang Wang

**Affiliations:** ^1^ School of Bioscience and Technology, Chengdu Medical College, Chengdu, China; ^2^ National Engineering Research Center for Biomaterials, Sichuan University, Chengdu, China; ^3^ Department of Cell and Chemical Biology, Leiden University Medical Center, Leiden, Netherlands; ^4^ Department of Abdominal Oncology, West China Hospital, Sichuan University, Chengdu, China

**Keywords:** FVII-Fc immunoconjugate, intramuscular gene expression, L/E technique, tumor immunotherapy, gene therapy

## Abstract

Tissue factor (TF) has been confirmed to be specifically expressed by vascular endothelial cells (VECs) in solid tumors and certain types of malignant tumor cells. Coagulation factor VII (FVII) can specifically bind to TF with high affinity, so the FVII-TF interaction provides an ideal target for tumor therapy. Expression of proteins in skeletal muscles is a simple and economical avenue for continuous production of therapeutic molecules. However, it is difficult to treat solid tumors till now due to the limited number of therapeutic proteins produced by the intramuscular gene expression system. Herein, we strived to explore whether anti-tumor effects can be achieved *via* intramuscular delivery of a plasmid encoding a FVII-guided immunoconjugate (Icon) molecule by a previously established Pluronic L64/electropulse (L/E) technique. Our study exhibited several interesting outcomes. 1) The mouse light chain of FVII (mLFVII) molecule could guide red fluorescent protein (RFP) to accumulate predominantly at tumor sites in a TF-dependent manner. 2) Intramuscular expression of mLFVII-hFc (human IgG1 Fc) Icon could significantly inhibit the growth of both liver and lung cancers in nude mice, and the inhibition extent was proportional to the level of tumor-expressed TF. 3) The number of blood vessels and the amount of blood flow in tumors were significantly decreased in mLFVII-hFc Icon-treated mice. 4) This immunotherapy system did not display obvious side effects. Our study provided an efficient and economical system for tumor immunotherapy by targeting both blood vessels and tumor cells. It is also an open system for synergistic therapy by conveniently integrating other anticancer regimens.

## Introduction

Tissue factor (TF), a 47-kDa membrane-bound receptor (1, 2), has been used primarily as the target molecule for specific tumor therapy, because TF is specifically expressed in tumor vascular endothelial cells (VECs) and some types of malignant tumor cells (3–6). As a natural ligand of TF, blood coagulation factor VII (FVII), which consists of a light chain (LFVII) and a heavy chain (7), can specifically bind to TF with high affinity (8). The LFVII is responsible for targeting and binding TF, while the heavy chain is in charge of coagulation function (9). In general, mouse LFVII (mLFVII) can bind tightly to both human and mouse TF, while human LFVII can only bind to human TF (10). Therefore, mLFVII may function as the guide molecule in tumor targeting therapies both in human and mouse systems. For safety concerns, mLFVII was entirely eliminated the coagulation function by deleting the heavy chain and introducing a point mutation (L341A) (11).

IgG is the main class of immunoglobulin produced during an immune response against foreign antigens and generally abundant in the circulation (12). The protective functions of IgG are mainly determined by its two functional domains: the Fab domain contributes to highly specific antigenic recognition and the Fc domain provides the IgG effector activity. Generally, Fc-mediated effects can be activated through the specific interactions with the Fc receptors (FcRs) that are widely expressed on some effector leukocyte types, such as phagocytic leukocytes and NK cells, resulting in the antibody-dependent cellular toxicity (ADCC) and complement-dependent cytotoxicity (CDC) (13). Based on these prominent characteristics, a fusion protein can be constructed, which retains the Fc domain of IgG and replaces the Fab domain with specific molecules as targeting unit. The mLFVII-Fc fusion protein, a kind of immunoconjugate (Icon), was designed and exhibited significantly suppressive effects on many solid tumors *via* intravenous or intratumoral injection of adenoviral vector encoding the Icon (11, 14, 15).

Skeletal muscle has been identified as an ideal tissue to obtain exogenous genes and secrete therapeutic proteins such as interleukin (IL)10 (16), erythropoietin (EPO) (17–19), or tumor necrosis factor (TNF)-α soluble receptor (20) *etc.* However, it is still a big challenge to treat solid tumors *via* intramuscular expression of plasmid-encoded therapeutic proteins, as the expression level has been the critical bottleneck in such trials. Many determinative factors were related to the protein expression level, including DNA delivery materials, DNA transfer methods and gene regulatory units, *etc.* A kind of amphiphilic triblock copolymers, which are unable to interact with DNA, can improve the plasmid DNA transfer efficiency into skeletal muscles (21). We previously revealed that, among these copolymers, Pluronic L64 not only enhanced the permeability of biomembranes by generating structural disturbance and pore formation, but also facilitated a rapid escape of the translocated complex from the endosome/lysosome compartments and caused a rapid dissociation of DNA from the complex (22). In addition, we established a novel intramuscular gene delivery method by combining Pluronic L64 and low-voltage electropulse. This chem-physical L64/electropulse (L/E) method displayed outstanding efficiency and good biocompatibility for gene delivery into skeletal muscles of mice (23).

The anti-angiogenesis strategy has been used for many years in tumor therapy. But increasing number of anticancer studies reported that the effects of anti-angiogenesis therapy alone are limited (24). In contrast, the synergistic therapies are more effective. Meanwhile, application of NK cells for tumor immunotherapy is presenting more and more attractive outcomes (25). To this end, we will construct a plasmid mLFVII-hFc to express a fusion protein. mLFVII serves as the guiding part for targeting tumor VECs as well as TF^+^ tumor cells. The effector part is the Fc domain of human IgG1 (hFc), which can activate the ADCC and CDC effects. The plasmid will be delivered into skeletal muscle cells using the L/E method, and the mLFVII-hFc Icon will be expressed and secreted into the blood circulation. It is an attractive anticipation that whether this system is able to continuously produce enough immunoconjugates to treat solid tumors by damaging tumor blood vessels and killing tumor cells.

## Materials and Methods

### Plasmid Construction

The plasmids were constructed based on the basic vectors pSecTag2 A (Invitrogen, Carlsbad, CA, USA) and pSC (constructed by our lab) (23). To construct pSCIgκ-mLFVII plasmid, mLFVII was amplified (forward primer mLFVII-F 5’-CCCAAGCTTGGCCAACTCACTCCTGGAGGAGCT-3’ and reverse primer mLFVII-R 5’-CGGGATCCGCGGCCTTGGCGGCTGCT-3’) from pcDNA-mLFVII (constructed by our lab) and inserted into pSecTag2 A plasmid within *Bam*HI/*Hin*dIII sites, thereby generating pSecTag2A-mLFVII. Then the Igκ-mLFVII fragment which contains the signal peptide of Igκ was digested by *Mlu*I/*Apa*I restriction enzymes from pSecTag2A-mLFVII and inserted into pSC to generate pSCIgκ-mLFVII. The gene of red fluorescent protein E2-Crimson was amplified (forward primer E2-F 5’-GTCGATATCGATAGCACTGAGAACGTCATCAAG-3’ and reverse primer E2-R 5’-TCGAGCGGCCGCTACTGGAA-3’) from pSC-E2 (constructed by our lab) (23) and inserted into pSCIgκ-mLFVII within *Not* I/*Eco*RV sites to generate pSCIgκ-mLFVII-E2. Similarly, pSCIgκ-mLFVII-Fc was constructed by cutting hFc from pcDNA-hFc (constructed by our lab) and inserting it into pSCIgκ-mLFVII within *Not*I/*Eco*RV sites.

### Cell Strains

Human hepatocellular carcinoma cell strain (SK-Hep-1), normal liver cell strain (L02) and human lung carcinoma cell strains (A549 and NCI-H292) were cultured in RPMI 1640 medium (Invitrogen, Carlsbad, CA, USA) + 10% fetal bovine serum FBS (Hyclone, Invitrogen). The stable SK-Hep1-EGFP strain which expresses the enhanced green fluorescent protein (EGFP) was constructed by transfecting the cells with pcDNA-EGFP plasmid (constructed by our lab) and selected with G418 antibiotic (Invitrogen).

### Flow Cytometry

For flow cytometry, about 1 × 10^6^ cells were collected and centrifuged at 1200 rpm/min for 10 min. The supernatant was discarded and mixed with FITC-labeled anti-tissue factor (TF) antibody (1:100 dilution, Shanghai Yanjing Biochemical Reagent Co. LTD, China) and incubated in dark for 30 min at 4°C. After being rinsed with PBS, the samples were fixed with 1% paraformaldehyde and analyzed by a Flow Cytometer (Beckman).

### Immunofluorescence Staining

The confluence of cells in the dish was kept between 20% and 30%, thereby allowing the cells to separate individually. The cell culture medium was discarded and cells were rinsed with PBS for three times, followed by incubating with FITC-labeled anti-TF antibody (1:100 dilution) in a 5% CO_2_ incubator at 37°C for 1 hour. Finally, cells were rinsed twice with PBS and imaged by confocal microscope (Leica Microsystem, Germany).

### Establishment of Xenograft Tumor Model in Nude Mice

Animals were treated in compliance with the relevant rules and guidelines, and the animal experimentations were permitted by the ethics committee of Sichuan University. 5-week-old male nude mice were purchased from Dashuo biotechnical company of Chengdu, China. After one-week adaptive period, 100 µL tumor cell suspension (1 × 10^6^ cells) was subcutaneously inoculated into the right rear flank of each nude mouse.

### Histological Section Preparation and Analysis

Immunohistochemistry staining for CD34 and α-SMA was performed on formalin-fixed, paraffin-embedded tumor sections. After deparaffinization and rehydration, endogenous peroxidase was blocked in 3% H_2_O_2_, and then sections were treated with a citrate buffer at 100°C in an oven for 60 min to allow antigen retrieval. The slides were then incubated overnight with monoclonal anti-CD34 antibody or anti-α-SMA antibody. EnVision™ system (Dako, Carpinteria, CA, USA) was used for secondary antibody detection and the reactions were visualized with diaminobenzidine.

To detect liver damage, livers were separated from mice after 35 days of treatment and fixed in 4% fresh neutral paraformaldehyde solution for 48h, and dehydrated subsequently. Well-dehydrated samples were embedded in paraffin, sliced in sections with 4 µm thickness. The sections of specimens were photographed by a microscope after staining with hematoxylin and eosin.

### Pluronic L64/Electropulse Gene Delivery Method

Pluronic L64 (L64; Sigma-Aldrich, St Louis, MO, USA) solution was diluted to 0.2% (w/v) using saline, mixed with equal volume of plasmid DNA (pDNA) and kept for 5 min at room temperature before injection. Each side of the TA muscle in a mouse was injected with 30 μL saline containing 30 μg pDNA/L64 (0.1%) mixture using a 29-gauge BD Ultra-Fine insulin syringe (BD, Franklin Lakes, NJ, USA). The injected site was approximately in the middle of a TA muscle and the injection time was 2–3 s. An hour after the injection, a clinically applied SDZ-V Nerve and Muscle Stimulator was used for electrotransfering. The electropulse parameters were: 5 Hz, intensity level of 3 and stimulation duration as 3 min. In order to increase the therapeutic effect of mLFVII-Fc, each mouse was injected with a therapeutic or control plasmid once a week.

### Blood Flow Measurement

The blood flow perfusion of subcutaneously tumor in nude mice 40 days after the plasmid injection was detected by Laser Speckle Blood Flowmetry (Periflux System 5,000, Perimed, Stockholm, Sweden). The perfusion rate in a tumor was normalized to that in the normal area near the tumor at same mouse.

### Fluorescent Protein Signal Imaging

For EGFP detection *in vivo*, the mice were anesthetized with 300 mg kg^-1^ chloral hydrate and imaged by the *in vivo* imaging system (CRI Maestro, Boston, MA, USA). Then the mice were irradiated by the blue light and scanned at 510 nm at room temperature to obtain the emitting green light. For RFP (E2-Crimson) detection in different xenograft tumors and different organs, each mouse heart, liver, spleen, lung, kidney and tumor were collected for the fluorescence imaging at 650 nm after the yellow light excitation.

### Blood Routine Examination and Serum Biochemical Test

BALB/c and nude mice were transferred with 30 μg pSCIgκ-mLFVII-Fc plasmid into TA muscle by L/E method as described above. The blood of each mouse was collected at the fifth or tenth day after first injection, respectively. The serum was separated from blood by centrifugation at 3000 rpm for 10 min at 4°C. The blood routine examinations were performed using the 7020 Automatic Analyzer (Hitachi, Japan) The sera biochemical tests were analyzed using the Analyzer Medical System AUTOLAB (AMS, Italy).

### Statistical Analysis

Data were expressed as the mean ± s.d. The comparisons among different groups were made using Student’s t-test to calculate two-tailed distribution and unequal variances. A value of P < 0.05 was considered significant difference, and P < 0.01 as highly significant difference.

## Results

### Clinical Survival Analysis of Tissue Factor Expression in Liver and Lung Carcinoma

**Figure 1:**
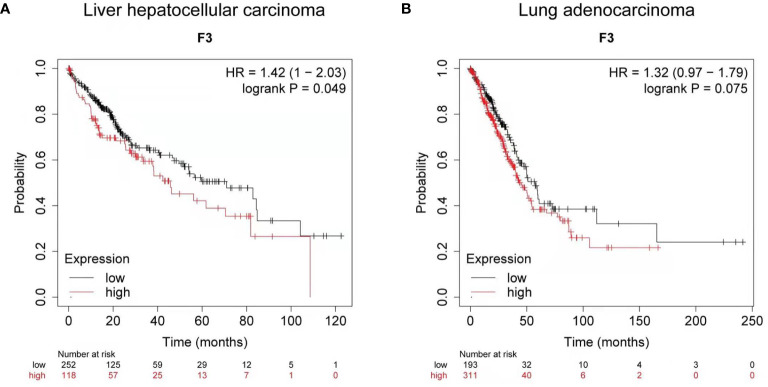
Clinical survival analysis of tissue factor expression in liver and lung carcinoma. **(A)** Kaplan-Meier survival curve for the overall survival (OS) of tissue factor (TF/F3) in liver hepatocellular carcinoma patients (n = 370). **(B)** Kaplan-Meier survival curve for OS of F3 in lung adenocarcinoma patients (n = 504). Data obtained from the Kaplan-Meier plotter database.

Using Kaplan-Meier clinical databases we found that tissue factor (TF/F3) is highly expressed in liver and lung carcinoma, which indicates TF can be a good target molecule for the liver and lung cancer treatment. The overall survival curves of 370 liver hepatocellular carcinoma patients **(** **Figure 1** **)** and 504 lung adenocarcinoma patients **(** **Figure 1** **)** showed that higher levels of tissue factor correlate with poor prognosis (26, 27). Therefore, developing a specific and efficient method to target TF for immunotherapy in liver and lung cancer will provide a promising regimen to prolong the survival of the liver and cancer patients.

### Validation of mLFVII’s Ability to Target SK-Hep-1 Cells With Highly Expressed TFs

**Figure 2:**
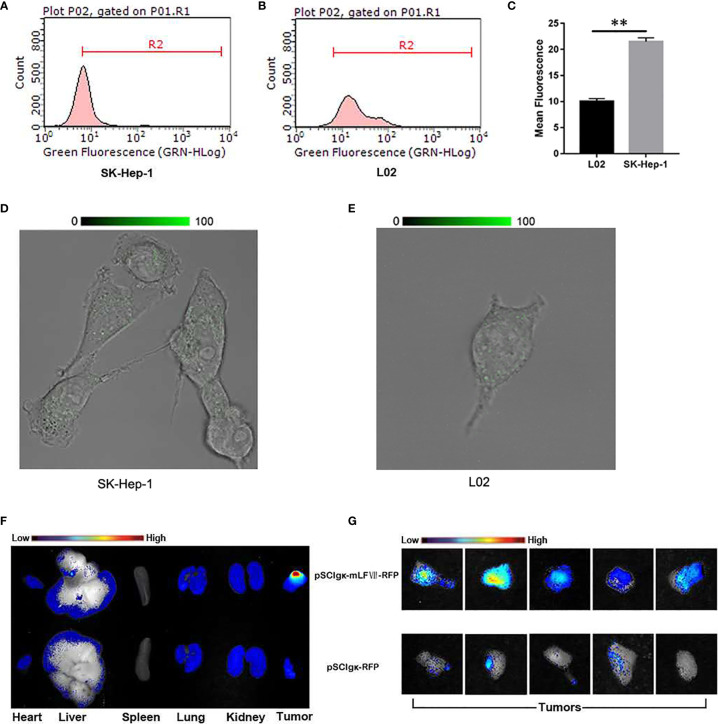
Analysis of the targeting ability of mLFVII on SK-Hep-1 cells and xenograft nude mice. **(A, B)** Flow cytometry histograms of the expression levels (presented as green fluorescence signals) of TF in SK-Hep-1 and L02 cell lines and **(C)** Quantification of TF expression level by mean fluorescence using Image Pro-Plus (IPP) software, **P < 0.01. **(D, E)** Representative immunofluorescent staining images of TF expression in SK-Hep-1 and L02 cells, respectively. Original magnification: 400 ×. **(F)** Representative *ex vivo* images of red fluorescent protein (RFP) signal in the heart, liver, spleen, lung, kidney and tumor of SK-Hep-1 xenograft mice after injection of pSCIgκ-RFP or pSCIgκ-mLFVII-RFP plasmid, respectively, n = 5 per group, and **(G)** comparison of locally enlarged RFP fluorescent signals at different tumor sites.

To study the targeting ability of mLFVII to TF, the expression levels of TF were compared between human hepatocellular carcinoma cell strain SK-Hep-1 and normal liver cell strain L02 by using flow cytometry **(** **Figures 2A–C** **)** and immunofluorescent staining **(** **Figures 2D, E** **)**. The results indicated that TF level on SK-Hep-1 was more than twice that of L02 cells. When the plasmid pSClgκ-mLFVII-RFP was transferred into the tibialis anterior (TA) muscles of SK-Hep-1-xenograft nude mice using the L/E method, the expressed fusion proteins mLFVII-RFP specifically targeted and gathered at the tumor sites and emitted strong red fluorescent signal. In contrast, the RFP alone, encoded by the plasmid pSClgκ-RFP, were not detected in any major organs and tumors **(** **Figures 2F, G** **)**. The results demonstrated that mLFVII was able to act as a guide component in the fusion protein, leading the effector protein to target and accumulate at tumor sites with high level of TF expression.

### Therapeutic Effect of mLFVII−hFc Icon on the SK-Hep1-EGFP Xenografts

**Figure 3:**
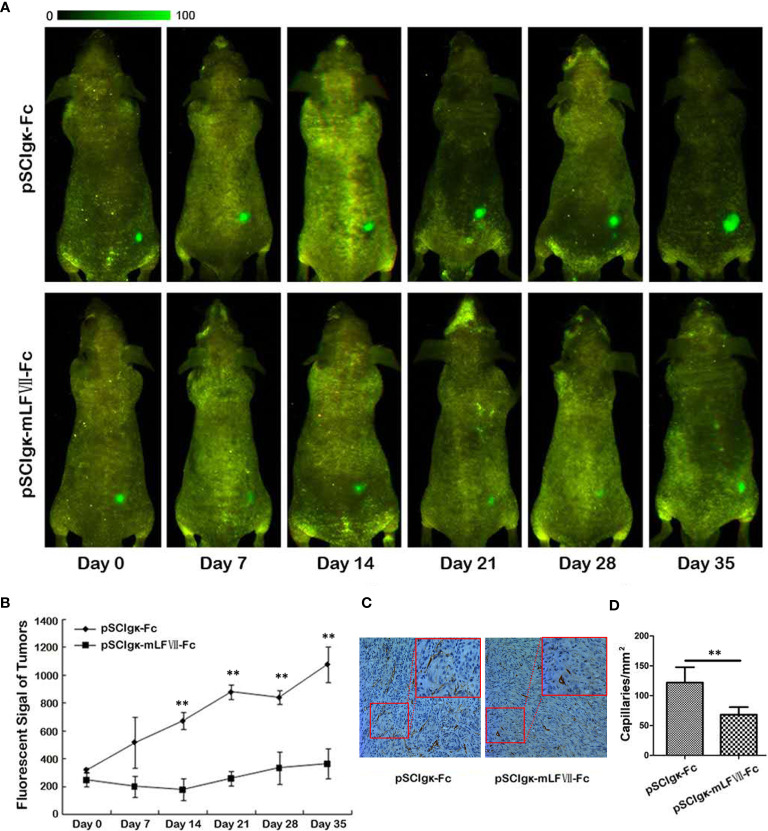
Therapeutic effect on EGFP-expressing SK-Hep-1 xenografts in nude mice. **(A)** Representative *in vivo* images of the EGFP-expressing SK-Hep-1 xenografts on days 0, 7, 14, 21, 28 and 35 after injection of pSCIgκ-Fc or pSCIgκ-mLFVII-Fc plasmid, respectively, and **(B)** quantitative analysis of the fluorescence intensity of the tumors using Image Pro-Plus software (IPP). n = 6 per group. **(C)** Representative immunohistochemical staining images of CD34 in tumors from different groups. Original magnification: 200×. **(D)** Quantification of the capillary density by counting the CD34^+^ fibers from five fields of view per slide at ×200 magnification. Capillary density was represented as capillaries per square millimeter. **P < 0.01.

To test the *in vivo* tumor growth inhibition effects of the mLFVII-hFc Icon, the stable cell strain SK-Hep1-EGFP which expresses the enhanced green fluorescent protein (EGFP) was constructed. When the plasmid pSClgκ-mLFVII-Fc or pSClgκ-Fc was intramuscularly delivered into the TA muscles of the SK-Hep1-EGFP-xenograft nude mice, the *in vivo* fluorescence intensity was continuously measured at different time points. The results showed that the signals in mLFVII-hFc-treated tumors increased slowly, whereas the signals in hFc-treated tumors quickly strengthened, indicating a significant inhibition of tumor growth by the mLFVII-hFc Icon treatment **(** **Figures 3A, B** **)**. In some mLFVII-hFc-treated tumors, the signals became weaker or even disappeared. Furthermore, the capillary density in the tumor was measured using anti-CD34 and anti-α-SMA immunohistochemical staining. The results demonstrated that the capillaries in mLFVII-hFc-treated tumors were significantly attenuated compared with those in hFc-treated tumors, confirming the anti-angiogenesis efficacy of mLFVII-Fc Icon **(** **Figures 3C, D** **)**. Therefore, it demonstrated that intramuscular injection of pSClgκ-mLFVII-Fc plasmid could effectively inhibit the growth of the TF^+^ tumors as well as the neovascularization in the tumors.

### Correlation Analysis of mLFVII Targeting Performance and TF Expression Level in Two Different Lung Cancer Cells

**Figure 4:**
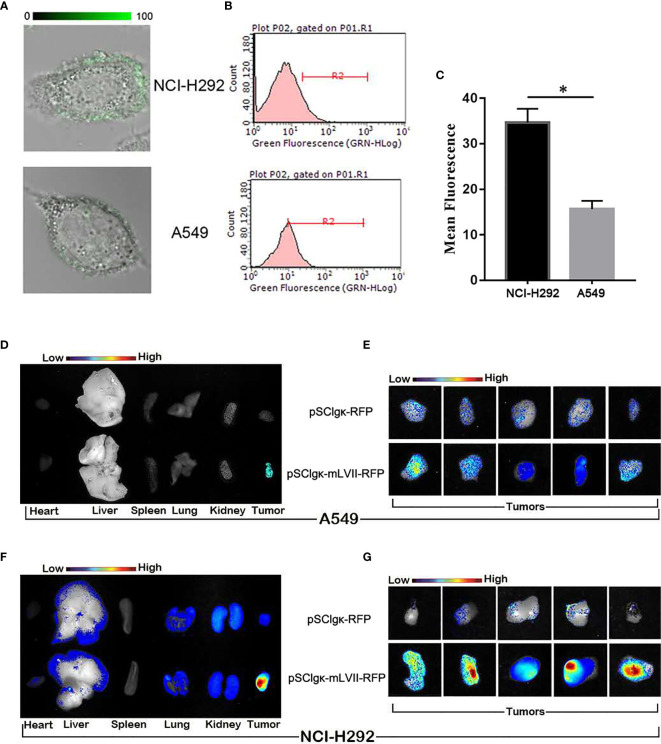
*In vitro* and *in vivo* analysis of the targeting tendency of mLFVII to TF level. **(A)** Representative immunofluorescent staining images of TF expression in NCI-H292 and A549 cells, respectively. Original magnification: 400×. **(B)** Flow cytometry histograms of the expression of TF in NCI-H292 and A549 cells, and **(C)** quantitative analysis of the expression level of TF by mean fluorescence using Image Pro-Plus (IPP) software. *P < 0.05. **(D)** Representative *ex vivo* images of the RFP signal in the heart, liver, spleen, lung, kidney and tumor after injection of pSCIgκ-E2 or pSCIgκ-mLFVII-E2 in A549 or **(F)** NCI-H292 xenograft mice, respectively. **(E)** Representative *ex vivo* images of locally enlarged RFP fluorescent signals in A549 or **(G)** NCI-H292 xenograft tumors, respectively.

It was found that mLFVII could target the highly TF-expressing tumor (SK-Hep-1), but whether the mLFVII targeting performance was consistent with the TF expression level on tumor cells need to be further studied. Two strains of lung cancers, NCI-H292 and A549, have been used to firstly detect the endogenous TF expression. Both immunofluorescent staining and flow cytometry results showed that the TF level in NCI-H292 cells are significantly higher than the level in A549 cells **(** **Figures 4A, C** **)**. Plasmid pSClgκ-RFP or pSClgκ-mLFVII-RFP was transferred into TA muscles of A549 or NCI-292 xenografted nude mice using L/E method, respectively. *Ex vivo* imaging analysis showed that the RFP with mLFVIIs could efficiently gather in tumor regions, whereas the RFP alone could not **(** **Figures 4D, G** **)**. More importantly, the RFP fluorescent signals in NCI-H292-xenografts were obviously stronger than those in A549-xenografts, corresponding to their respective TF expression levels **(** **Figures 4D, G** **)**. So, the results indicated that mLFVII-guided fusion proteins could target and gather in tumor sites in a TF-level dependent manner.

### Therapeutic Efficacy of mLFVII−Fc on A549 and NCI-292 Xenografts in Nude Mice

**Figure 5:**
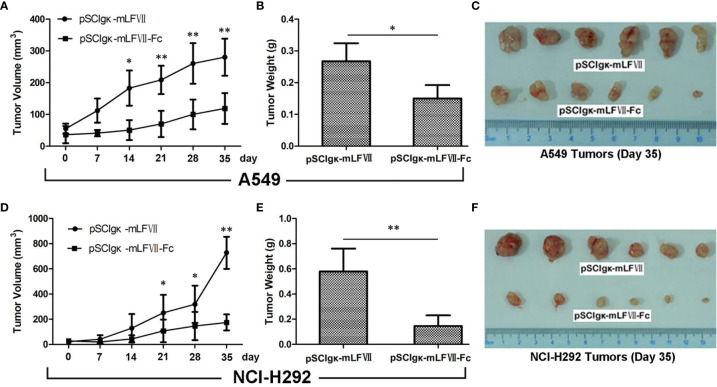
Therapeutic efficacy of mLFVII−Fc Icon on A549 and NCI-H292 xenografts in nude mice Tumor volumes at different days **(A, D)**, tumor weight **(B, E)** and size **(C, F)** at day 35 after plasmid injection. n=6 per group, *p < 0.05, **p < 0.01.

The plasmid pSClgκ-mLFVII-Fc exerted a significant inhibitory effect on both types of lung tumors, as confirmed by continuous tumor volume measurements **(** **Figures 5A, D** **)** and the final tumor weights **(** **Figures 5B, E** **)**. More importantly, for NCI-H292-xenografts which possess higher TF expression, the final average tumor weight in pSClgκ-mLFVII-treated group was 3 times more than that in pSClgκ-mLFVII-Fc-treated group **(** **Figure 5E** **)**. In comparison, for A549-xenografts which possess lower TF expression, the ratio was about 1.8 **(** **Figure 5B** **)**. The results demonstrated that mLFVII-Fc Icon could inhibit tumor growth in a TF-level dependent manner.

### The Effect of mLFVII−Fc on Blood Flow and Vascular Density in Tumors

**Figure 6:**
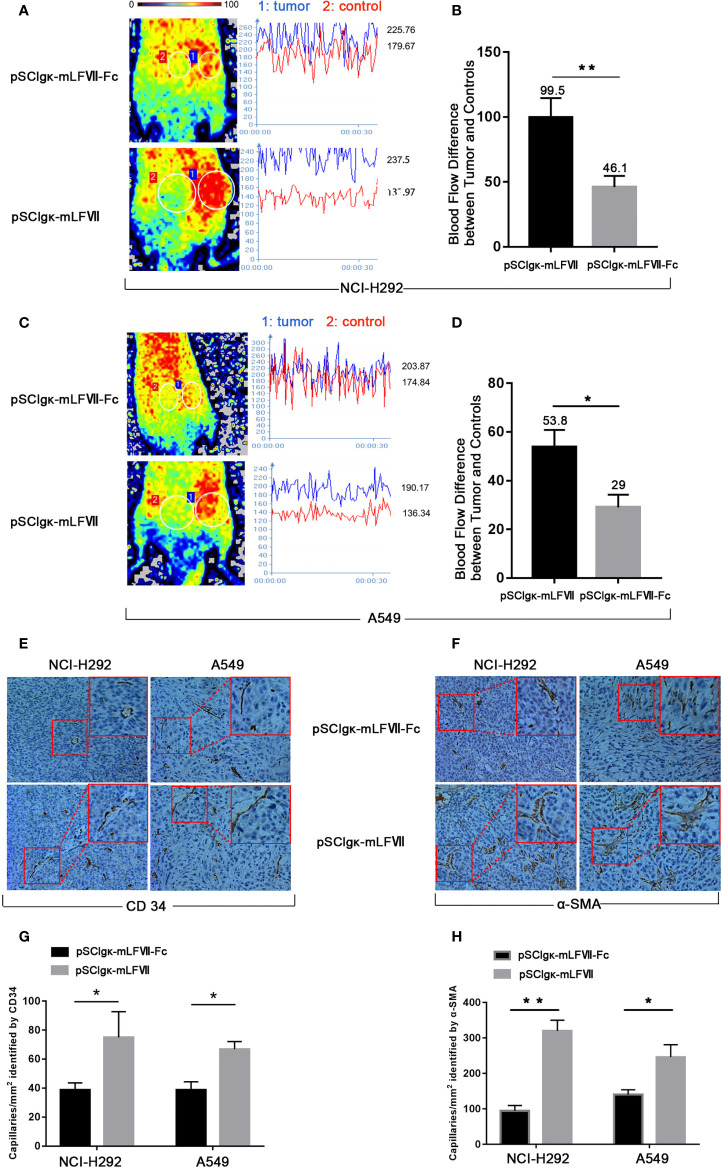
Analysis of the blood flow and capillary density in NCI-H292 and A549 xenografts. Representative images and quantification analysis of the blood flow perfusion in NCI-H292 **(A, B)** or A549 **(C, D)** xenografts in nude mice 40 days after plasmid injection. Tumor: the measured area of tumor xenograft. Control: the measured normal area adjacent to the tumor. n=6 per group. Representative immunohistochemical images of anti-CD34-stained **(E)** or anti-α-SMA-stained **(F)** in NCI-H292 or A549 xenografts. Quantification of the capillary density by counting the CD34^+^ **(G)** or α-SMA^+^ **(H)** fibers from five fields of view per slide at × 200 magnification, n=6 per group, *p<0.05, **p<0.01.

As TFs specifically expresses in tumor vascular endothelial cells, we measured the blood perfusion of A549 or NCI-H292 xenografts to evaluate the therapeutic effects of mLFVII-Fc on tumor blood vessels. After deep anesthesia, the nude mice were placed in the prone position on the Laser Speckle Blood Flowmetry test platform. The tumor inoculated on the right buttocks of nude mice was selected as the blood flow detection area (circled by white), and the uninoculated area on the left buttocks of the same nude mice was selected as the control area (circled by white) **(** **Figures 6A, C** **)**, so as to minimize the variability due to individual basal perfusion. As shown in **Figures 6A, C** the blood flows in tumor regions were still much higher than those in the adjacent normal sites after the pSClgκ-mLFVII application. However, the differences were significantly decreased after pSClgκ-mLFVII-Fc plasmid administration, indicating that the blood flows in tumors were obviously reduced after the mLFVII-Fc Icon treatment, especially in NCI-H292 xenografts **(** **Figures 6A–D** **)**. Meanwhile, according to the immunohistochemical staining results of CD34 and α-SMA, pSClgκ-mLFVII-Fc plasmid treatment significantly decreased the density of blood vessels in both A549 and NCI-H292 xenografts **(** **Figures 6E–H** **)**. These results suggested that intramuscular injection of pSClgκ-mLFVII-Fc plasmid could effectively reduce the blood supply in the tumors, and the reduction was most likely due to the destruction of tumor blood vessels.

### *In Vivo* Safety Analysis of pSClgκ-mLFVII-Fc Treatment

**Figure 7:**
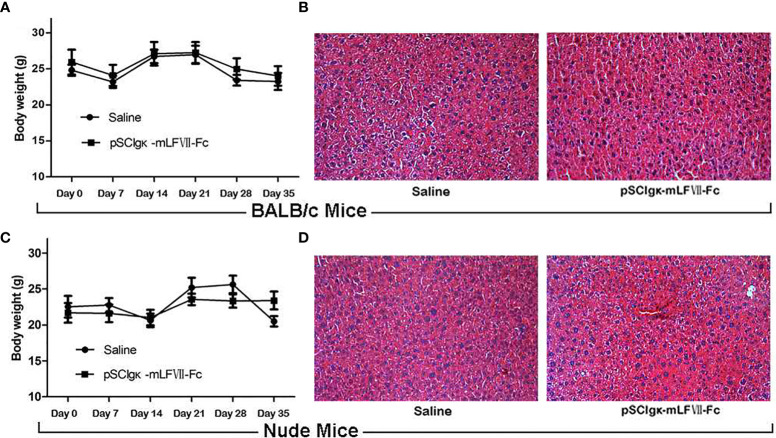
Safety assay of BALB/c and nude mice after pSCIgκ-mLFVII-Fc treatment. Average body weights of BALB/c **(A)** or nude mice **(C)** at different days after injection. n=6 per group. Representative liver H&E staining images of BALB/c **(B)** or nude mice **(D)** 35 days after treatment. n=6 per group, Original magnification: 200×.

As shown in **Figures 7A, C**, the body weights of both BALB/c and nude mice did not show any significant difference at every time points between the pSClgκ-mLFVII-Fc group and saline group (P>0.05). Hematoxylin eosin (HE) staining results of liver also demonstrated no significant difference between these two groups **(** **Figures 7B, D** **)**. Furthermore, routine blood examination and blood biochemical analysis (including liver function, kidney function and other indicators) also indicated that the pSClgκ-mLFVII-Fc system had no influence on the various vital indicators **(** **Tables 1**–**4** **)**. Taken together, the data confirmed that pSClgκ-mLFVII-Fc did not trigger any significant side effects and was safe for *in vivo* applications.

**Table 1:** Serum biochemical test of BALB/c mice.

Items		control	p(5)	p(10)
liver function	ALT (U/L)	49 ± 4.58	51 ± 70.33	52.67 ± 9.07
	AST (U/L)	139 ± 11.53	149.33 ± 24.22	165.33 ± 8.33
	ALP (U/L)	258.67 ± 40.27	220 ± 1.81	247 ± 33.42
	ALB (U/L)	25.8 ± 0.98	25.8 ± 5.34	26.63 ± 1.21
	TBIL (μmol/L)	10.22 ± 0.99	11.92 ± 1.66	28.05 ± 5.34
renal function	BUN (mmol/L)	6.27 ± 0.07	5.36 ± 13.82	7.86 ± 1.05
	CRE (μmol/L)	38 ± 0	36.67 ± 5.34	38 ± 4.36

**Table 2:** Serum biochemical test of nude mice.

Items		control	p(5)	p(10)
liver function	ALT (U/L)	47.33 ± 3.21	45.33 ± 3.51	58 ± 1.41
	AST (U/L)	156 ± 5.29	130.67 ± 8.96	160 ± 24.22
	ALP (U/L)	344 ± 25.06	260.5 ± 13.44	255.33 ± 1.81
	ALB (U/L)	30.57 ± 0.38	25.93 ± 0.46	27.13 ± 5.34
	TBIL (μmol/L)	11.69 ± 1.21	12.51 ± 3.21	31.32 ± 19.61
renal function	BUN (mmol/L)	5.31 ± 0	6.07 ± 0.85	3.04 ± 13.82
	CRE (μmol/L)	41 ± 0	43 ± 0	53.67 ± 7.03

ALT, alanine aminotransferase; AST, aspartate aminotransferase; ALP, alkaline phosphatase; ALB, albumin; TBIL, total bilirubin; BUN, blood urea nitrogen; CRE, creatinine.

**Table 3:** Routine blood test of BALB/c mice.

Items	control	p(5)	p(10)
WBC (10^9/L)	6.78 ± 0.83	6.09 ± 1.93	6. 12 ± 0.24
RBC (10^12/L)	10.5 ± 0.57	9.93 ± 0.6	9. 15 ± 1.01
HGB (g/L)	186.67 ± 7.64	170.33 ± 11.59	160.67 ± 18.77
HCT (L/L)	49.8 ± 2.33	45.95 ± 3.34	43.38 ± 4.08
MCV (fL)	47.33 ± 0.58	46.33 ± 0.58	47.33 ± 0.58
RDWcv (%)	18.7 ± 0.35	19.63 ± 0.23	18.33 ± 1.03
MCH (pg)	17.8 ± 0.26	17.17 ± 0.31	17.57 ± 0.29
MCHC (g/L)	375 ± 7.94	370.67 ± 6.03	370 ± 8.89
PLT (10^9/L)	459 ± 62.65	490 ± 55.15	456 ± 55.87
LY% (%)	71.83 ± 3.23	69.27 ± 13.8	74.8 ± 12.29
MI% (%)	2.23 ± 1.93	3.13 ± 2.66	2.8 ± 2.1
GR% (%)	25.9 ± 2.96	27.57 ± 12.42	22.37 ± 10.38
MPV (fL)	8.03 ± 0.21	6.97 ± 0.58	6.93 ± 0.4
PCT (%)	0.37 ± 0.05	0.39 ± 0.1	0.32 ± 0.06
LYM (10^9/L)	4.88 ± 0.76	4.34 ± 2.05	4.57 ± 0.67
MID (10^9/L)	0.14 ± 0.1	0.2 ± 0.16	0.17 ± 0.13
GRA (10^9/L)	1.75 ± 0.28	1.55 ± 0.41	1.38 ± 0.67

**Table 4:** Routine blood test of nude mice.

Items	control	p(5)	p(10)
WBC (10^9/L)	2.63 ± 0.21	4.42 ± 0.45	3.71 ± 0.23
RBC (10^12/L)	11.41 ± 0.25	9.48 ± 0.31	10.01 ± 0.2
HGB (g/L)	196.33 ± 7.02	163.67 ± 10.02	176 ± 10.15
HCT (L/L)	53.98 ± 1.48	45.1 ± 2.77	47.48 ± 2.32
MCV (fL)	47.33 ± 0.58	47.67 ± 1.53	47.33 ± 1.53
RDWcv (%)	21.4 ± 0.1	20.53 ± 1.8	20.37 ± 1.15
MCH (pg)	17.2 ± 0.2	17.27 ± 0.5	17.53 ± 0.68
MCHC (g/L)	363.33 ± 4.04	363 ± 0	370 ± 4.58
PLT (10^9/L)	579.67 ± 106.3	518.33 ± 69.83	452 ± 156.75
LY% (%)	48.67 ± 10.58	58.6 ± 9.97	61.7 ± 4.29
MI% (%)	5.93 ± 2.78	4.9 ± 0.69	6.5 ± 1.91
GR% (%)	45.4 ± 9.6	36.5 ± 9.66	31.8 ± 5.91
MPV (fL)	8.3 ± 0.2	7.23 ± 0.64	7.97 ± 1.19
PCT (%)	0.48 ± 0.09	0.38 ± 0.08	0.36 ± 0.09
LYM (10^9/L)	1.28 ± 0.29	2.56 ± 0.22	3.29 ± 1.84
MID (10^9/L)	0.16 ± 0.09	0.22 ± 0.04	0.24 ± 0.08
GRA (10^9/L)	1.19 ± 0.23	1.64 ± 0.61	1.18 ± 0.2

WBC, white blood cell count; RBC, red blood cell count; HGB, hemoglobin concentration; HCT, hematocrit; MCV, mean corpuscular volume; RDW, red blood cell distribution width; MCH, average RBC hemoglobin content; MCHC, mean corpuscular-hemoglobin concentration; PLT, blood platelet count; LY, lymphocyte percentage; MI, intermediate cells percentage; GR, neutrophile granulocyte percentage; MPV, mean platelet volume; PCT, thrombocytocrit; LYM, lymphocyte count; MID, intermediate cells count; GRA, neutrophile granulocyte count; p(5), routine blood was detected after the 5th day of pSCIgκ-mLFVII-Fc plasmid injection; p(10), routine blood was detected after the 10th day of pSCIgκ-mLFVII-Fc plasmid injection. p(5), tests were performed at the 5^th^ day after pSCIgκ-mLFVII-Fc plasmid injection; p(10, tests were performed at the 10^th^ day after pSCIgκ-mLFVII-Fc plasmid injection.

## Discussion

In this study, we confirmed that the mLFVII could guide the fusion protein mLFVII-RFP to target and gather at the tumor sites in xenograft mice **(** **Figures 2F, G** **)**, and this targeting ability was positively correlated with the TF expression level of tumor **(** **Figures 4D–G** **)**. The results also illustrated that the muscle cells were able to “produce” enough fusion proteins for *in vivo* detection **(** **Figures 2F, G**, **4D–G** **)**. These “self-produced” proteins significantly suppressed the growth of solid tumors **(****Figures 3B**, **5** **)**, decreased the numbers of capillaries **(** **Figures 3C, D**, **6C–F** **)** and reduced the blood flows in the tumors (**Figures 6A, B**). The therapeutic effects were found to be proportional to the TF levels of tumors (**Figures 4** ****–**6**), indicating that this treatment system requires high expression of TF to be effective. In addition, our treatment system was found to be safe and feasible, without significant toxic and side effects **(** **Figure 7** and **Tables 1**–**4** **)**, and thus exhibited promising potential for applications in more clinical trials.

The key to tumor immunotherapy lies in identifying molecules that are specifically expressed in tumor cells or tumor microenvironment (TME) including immune cells, cancer associated fibroblasts (CAFs) and vascular endothelial cells (VECs), whereas these molecules are restricted or not expressed in the normal tissues (28). Thus, these molecules can be ideal targets for developing immunotherapeutic drugs to treat tumors with minimum side effects. TF is such a molecule that has been reported to be expressed by VECs in the vasculature of solid tumors but not of normal tissues (3–6). It is also expressed by some types of tumor cells (3–6). A number of studies have shown that TF can regulate angiogenesis under a variety of pathological conditions. For instance, TF acted as an angiogenesis-specific receptor in angiogenic VECs of the age-related macular degeneration (AMD) (29) pathological neovasculature of endometriosis (30) and solid cancers, including melanoma (11), lung cancer (31–33) as well as breast cancer (34–37). TF is also expressed in the tumor xenografts in mice (38). In summary, TF can be used as a highly specific targeting molecule for immunotherapy in various solid tumors.

In most traditional studies, protein drugs were either produced and purified *in vitro* and then injected into the body, or expressed by intravenously or intratumorally injected viral vectors. In comparison, the main feature of our system was to use the autologous skeletal muscle cells to generate and secrete fusion proteins with therapeutic functions. The plasmid-based intramuscular gene delivery/expression system is obviously cheaper, safer and more convenient. The decisive factor to make it applicable for gene therapy is to effectively deliver plasmids into muscle cells.

At present, the commonly used gene delivery methods are mainly divided into two categories: viral vectors and non-viral vectors (39). Viral vectors can provide a high gene transfer efficiency, but adverse immune responses and carcinogenic effects may drastically limit their potential clinical applications. Recently, recombinant adeno-associated virus (rAAV) vector, which has been considering as the safest and most promising vector for clinical applications, was found to cause “1,741 unique AAV integration events in genomic DNA in liver samples from six treated dogs and expanded cell clones in five dogs, with 44% of the integrations near genes involved in cell growth” (40). So, the potential genotoxicity of AAV is still a security risk. Non-viral vectors mainly include cationic lipids, polymer and electrical impulse mediated gene delivery methods, which have the advantages of low toxicity, low immunogenicity as well as lower cost (41). However, their gene delivery efficiency was found to be quite inferior to that of viral vectors. In previous study, we found that Pluronic L64, a neutral amphiphilic triblock copolymer, could significantly enhance the permeability of biomembranes by causing structural disturbance and pore formation in a concentration and time dependent manner. In addition, it also displayed a unique ability to facilitate the rapid escape of the transfection complex from the endosomes and promoted the dissociation of the complex (22). We found that L64-mediated stable HIF-1α expression in muscles showed the capability to induce therapeutic angiogenesis in mouse hindlimb ischemia (42). We further established a more effective method by combining L64 and low-voltage electropulse. In this method, pre-injection of L64 may increase the permeability of *in situ* muscle cells and the subsequent electric field power may drive the negatively charged plasmids to translocate into the cells (23). Our study showed that this L/E method can efficiently transfer pSClgκ-mLFVII-Fc plasmid into skeletal muscle cells and produce mLFVII-Fc Icon for solid tumors treatments.

In addition to the distinctive feature of intramuscular gene delivery/expression, the system reported here combined two promising aspects. First, it employed the ADCC and CDC effects, which were mediated by NK cells and phagocytic leukocytes. For tumor immunotherapy, the predominant methods usually utilize T cells to kill tumor cells, including antibody-based immune checkpoint blockage and cell-based CAR-T therapy. Currently, NK cell-based therapies, such as CAR-NK, are becoming more and more important (43). So, it is an attractive and promising strategy to express both mLFVII-Fc Icon and immune checkpoint blockage antibodies in our system. Second, it mainly damaged already formed blood vessels in tumors due to its potent targeting ability on TF molecules specifically expressed by VECs in solid tumors. Anti-angiogenesis strategy has been applying to tumor therapy for many years. Vascular endothelial growth factor (VEGF) is the most commonly used target for anti-tumor angiogenesis. The functions of anti-VEGF and anti-VEGFR antibodies mainly depend on inhibiting the formation of new blood vessels in tumors (44). So, it is still feasible to simultaneously express anti-angiogenesis antibodies, such as anti-VEGF or anti-VEGFR antibodies, in our system. It may bring better outcomes. Therefore, our study offers an open system for conveniently integrating other tumor therapy avenues to develop more effective synergistic therapy regimens, without obviously increasing the cost.

## Data Availability Statement

The original contributions presented in the study are included in the article/supplementary material. Further inquiries can be directed to the corresponding authors.

## Ethics Statement

The animal study was reviewed and approved by the Institutional Committee on Animal Care of Sichuan University.

## Author Contributions

GW and ML designed the experiments. LM and GRW performed the experiments with support from SL. GW, ML, and FB analyzed the data. GW and LM wrote the paper. GW supervised the project. All authors contributed to the article and approved the submitted version.

## Funding

The work is supported the National Natural Science Foundation of China (No. 31971390, 81900564) and Chengdu Medical College Foundation (No. CYZ18-07).

## Conflict of Interest

The authors declare that the research was conducted in the absence of any commercial or financial relationships that could be construed as a potential conflict of interest.
